# Quality of maternity care and its determinants along the continuum in Kenya: A structural equation modeling analysis

**DOI:** 10.1371/journal.pone.0177756

**Published:** 2017-05-16

**Authors:** Patrick Opiyo Owili, Miriam Adoyo Muga, Bomar Rojas Mendez, Bradley Chen

**Affiliations:** 1 Institute of Environmental & Occupational Health Sciences, National Yang-Ming University, Taipei, Taiwan; 2 Department of Public Health, University of Eastern Africa, Baraton, Eldoret, Kenya; 3 College of Public Health and Nutrition, Taipei Medical University, Taipei, Taiwan; 4 International Health Program, National Yang-Ming University, Taipei, Taiwan; 5 Institute of Public Health, National Yang-Ming University, Taipei, Taiwan; University of Michigan Medical School, UNITED STATES

## Abstract

**Background:**

Improving access to delivery services does not guarantee access to quality obstetric care and better survival, and therefore, concerns for quality of maternal and newborn care in low- and middle-income countries have been raised. Our study explored characteristics associated with the quality of initial assessment, intrapartum, and immediate postpartum and newborn care, and further assessed the relationships along the continuum of care.

**Methods:**

The 2010 Service Provision Assessment data of Kenya for 627 routine deliveries of women aged 15–49 were used. Quality of care measures were assessed using recently validated quality of care measures during initial assessment, intrapartum, and postpartum periods. Data were analyzed with negative binomial regression and structural equation modeling technique.

**Results:**

The negative binomial regression results identified a number of determinants of quality, such as the level of health facilities, managing authority, presence of delivery fee, central electricity supply and clinical guideline for maternal and neonatal care. Our structural equation modeling (SEM) further demonstrated that facility characteristics were important determinants of quality for initial assessment and postpartum care, while characteristics at the provider level became more important in shaping the quality of intrapartum care. Furthermore we also noted that quality of initial assessment had a positive association with quality of intrapartum care (*β* = 0.71, *p* < 0.001), which in turn was positively associated with the quality of newborn and immediate postpartum care (*β* = 1.29, *p* = 0.004).

**Conclusions:**

A continued focus on quality of care along the continuum of maternity care is important not only to mothers but also their newborns. Policymakers should therefore ensure that required resources, as well as adequate supervision and emphasis on the quality of obstetric care, are available.

## Introduction

Maternal survival has been one of the most important developments and global health priorities in the past decades, exemplified by its adoption as the fifth Millennium Development Goals (MDG) [1,2] and subsequently the third Sustainable Development Goals (SDG) [3]. Nevertheless, the goal to reduce maternal mortality by three quarters by 2015 was the goal that lagged furthest behind its target among all [4]. There were still estimated over 300,000 preventable maternal deaths, at a rate of 830 deaths per day, in 2015 [5]. The majority of these maternal mortalities occur during delivery or shortly after [6]. This intrapartum and postpartum period is critical not only to mothers but also to their newborns. Out of the estimated 2.7 million preventable neonatal deaths in 2015, one million occurred during their first day of life [7]. Experts believe that the best strategy to reduce these intrapartum- and postpartum-related deaths is access to skilled birth attendant’s services [8,9].

However, improving access to delivery services does not always guarantee access to quality obstetric care and better survival. For instance, the Government of India launched in 2005 one of the largest conditional cash transfer programs in the world, Janani Suraksha Yojana (JSY), to encourage expectant mothers to obtain quality obstetric services in public or accredited private facilities [10]. Despite the significant increases in institutional deliveries, there is no strong evidence to support that the JSY program has contributed to improved maternal or neonatal survival [11,12]. Experiences from other countries have also demonstrated that improvement in utilization without concomitant increase in quality of care is inadequate [13–15]. The presumption that expansion in maternal service coverage would automatically lead to sustained reductions in maternal and neonatal deaths not only compromises our abilities to close the gap in women’s health, but also results in incredible inefficiency in the use of the already limited resources. Therefore, in recent years a more focused attention to quality of care for maternal and newborn care, particularly in low- and middle-income countries (LMICs) has been called for [15–17].

There are several critical challenges before effective interventions to improve quality of maternity care can become available. First and foremost is the lack of clarity on the definition of quality of maternity care [16,18–20]. Quality of care means different things to different people in various contexts [21]. The complexities and unpredictability of obstetric complications further presents unique difficulties in defining quality [18]. More recent studies have, in general, followed the Donabedian framework—structure, process and outcome, in conceptualizing quality of care [22]. However, even with a consensus on the definition of quality, measurement and monitoring of quality in obstetric services is anything but simple and straightforward. The structural aspects of quality are frequently measured in LMICs, but it is well recognized that the readiness of service provision or competence of health professionals does not ensure that mothers receive quality care [23]. Measuring maternal outcomes, such as fatality rate or mothers’ satisfaction as an alternative outcome indicator, is not satisfactory, either, because obstetric complications are unpredictable and may take place even with the best quality of care [8]. The measurement of process indicators, on the other hand, has been shown to best reflect the quality of care for mothers and newborns [24]. However, there is still no consensus on the gold standard of process quality indicators in LMICs. Some resort to “checklists” developed through expert opinions, but few of these are validated empirically [17]. Others emphasize the signal functions of emergency obstetric care including neonatal resuscitation [25], and yet such approach can also lead to inattention to the ability to provide quality routine maternal and neonatal care functions in a health facility [26,27]. Most recently, Tripathi and colleagues developed an expert-based index to measure process quality over the entire process from intake, intrapartum to immediate postpartum periods, with a focus on routine care, and most importantly, validated the index for application in sub-Saharan Africa [17]. The availability of such a tool presents great opportunities to assess the process of maternity care in LMICs, where such information is extremely limited.

Yet, development of programs that would enable improvement in the quality of care in LMICs still requires a thorough understanding of its determinants. This is especially important in LMICs where resources for quality improvement are rather limited given other competing needs. In addition to the challenge of defining quality measures, measuring quality along the continuum of care presents another challenge which increases the complexity in evaluating the quality of care. The term continuum of care has been used differently in various fields such as health-service administration, palliative care, biomedical care, mental health, public health/health promotion, and maternal, newborn and child health care, but it has generally been used to reflect the continuity between components of care [28–30]. In this study, continuum of care specifically refers to the entire clinical experience of a mother from the initial assessment before delivery to the postpartum care. Therefore, our study, following Trpathi et al.’s approach in measuring quality of obstetric services, explored the characteristics associated with the quality of initial assessment, intrapartum, and immediate postpartum and newborn care. Leveraging a nationally representative Service Provision Assessment (SPA) of Kenyan health facilities, our study not only examined the potential influences of different supply-side factors with the typical regression approach in prior studies, we also employed a structural equation modeling (SEM), which, in addition to a number of methodological advantages, allowed us to further explore the relationships along the continuum of care.

## Methods

### Data

**Fig 1:**
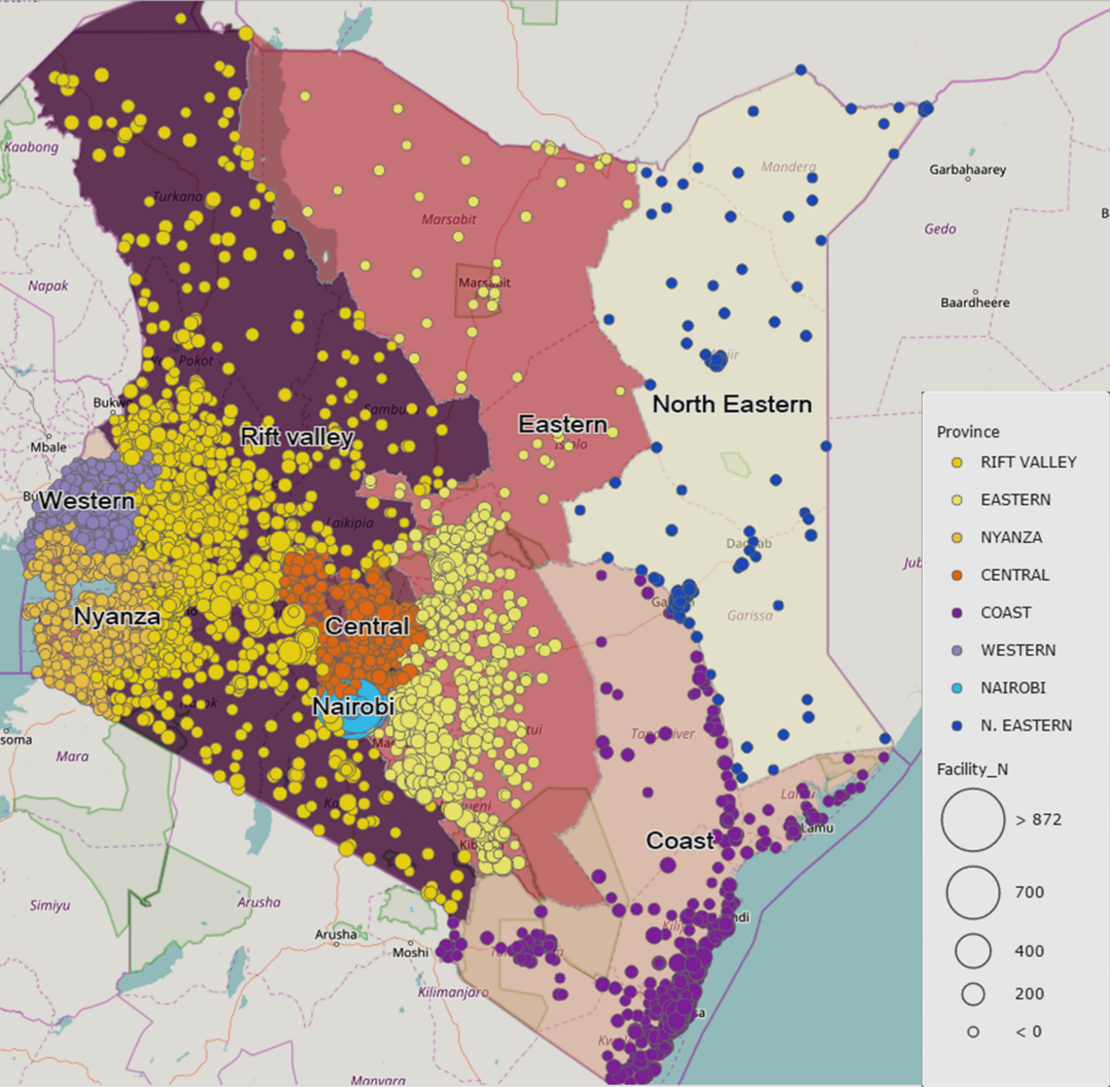
Distribution of health facilities in Kenya by provinces.

The 2010 SPA dataset of Kenya was used to explore our objectives. The SPA is a nationally representative health facility cross-sectional survey, and its complete details can be found elsewhere [31]. The survey data included availability of all necessary items in the facility for providers to offer quality delivery services as well as the providers’ information. Random sampling was used to select health facilities from the Master Facility List (MFL) of 6,192 operational public and private health facilities (see distribution of facilities across the country in Fig 1). The sampling strategy was designed to allow for representation of all levels of health care system and different managing authorities. A total of 703 facilities were sampled, representing roughly 11% of all health facilities in Kenya. In these facilities, health professionals who attended 627 routine delivery clients aged 15–49 were assessed for adherence to several signal functions and tracer items of delivery care that included intrapartum, newborn and immediate postpartum care. The signal functions used in our study are grounded on validated signal functions in sub-Saharan Africa (SSA) [17]. However, there were varying extent of missing data patterns in different indicators of the initial assessment and examination stage (221), intrapartum care (224), and newborn and immediate postpartum care (111). Moreover, the determinants of quality of care variables also had missing values as indicated herein: level of health facility (25), age (39), years of experience (39), and received incentives (39). After excluding the missing variable in any of the indicator used in our analyses, only 290 observations had complete data. Handling of missing data is described in the statistical analysis subsection.

### Measures

#### Quality of intrapartum, postpartum and newborn care

The validated and recommended signal functions and tracers of quality of intrapartum, newborn and postpartum care–suggested by experts in SSA [17] and listed in Table 1 –included 7 items during initial assessment and examination stage (i.e. asked if the woman experienced any danger sign, performed a general examination, took the temperature, took the blood pressure, took the pulse, washed hands before examination, and wore sterile gloves for vaginal examination), 7 items at the time of intrapartum care (i.e. explained all procedures, prepared uterotonic drug for active management of third stage labor, used partograph during labor, prepared newborn resuscitation equipment, correctly administered uterotonics, assessed integrity of placenta/membranes, and assessed for perineal/vaginal lacerations), and 6 items for newborn and immediate postpartum care (i.e. immediately dried the baby with towel, assessed newborn resuscitation effort and placed on mother’s abdomen skin-to-skin, tied or clamped the cord but not immediately after birth, took the mother’s vital signs 15 minutes after birth, palpated uterus 15 minutes after delivery, and assisted the mother to initiate breastfeeding within 1 hour). All the quality of care indicators were dichotomized. These quality measures reflect the minimum standards of obstetric care, irrespective of the type of health facilities where the delivery service is performed.

**Table 1:** Quality of facility-based delivery care, facility, provider and region characteristics, by health facility level.

		Health facility level, *n* (%_wt_)				
Variables		National/provincial *n* = 56	District [Sub] *n* = 163		Private^a^ *n* = 71	*P*-value
**Outcome indicators**						
*Initial assessment and examination*						
	Asked if experienced any danger sign	24 (51.2)	45 (27.1)		27 (37.4)	0.009
	Performed general examination	47 (87.0)	94 (59.5)		47 (65.4)	0.001
	Took temperature	31 (61.5)	61 (33.9)		43 (60.1)	< 0.001
	Took blood pressure	52 (94.7)	120 (71.9)		62 (86.3)	< 0.001
	Took pulse	48 (88.1)	87 (52.3)		54 (75.8)	< 0.001
	Washed hands before examination	17 (32.6)	46 (33.1)		29 (45.9)	0.244
	Wear sterile gloves for vaginal exam	56 (100.0)	160 (98.8)		71 (100.0)	0.425
*Intrapartum care*						
	Explained all procedures	36 (65.2)	104 (62.8)		50 (71.1)	0.549
	Prepared uterotonic drug for AMTSL	53 (97.7)	155 (96.4)		70 (99.3)	0.252
	Used partograph during labor	52 (94.9)	144 (87.0)		66 (94.7)	0.066
	Prepared newborn resuscitation equipment	26 (44.3)	48 (27.8)		43 (61.9)	< 0.001
	Correctly administered uterotonic	56 (100.0)	163 (100.0)		71 (100.0)	0.624
	Assessed integrity of placenta/membranes	42 (78.7)	95 (58.4)		43 (59.1)	0.026
	Assessed for perineal/vaginal lacerations	56 (100.0)	161 (98.8)		71 (100.0)	0.504
*Newborn and immediate postpartum care*						
	Immediately dried baby with towel	38 (82.2)	81 (49.7)		47 (63.5)	< 0.001
	Assessed NRE and placed skin-to-skin	52 (95.3)	102 (59.2)		40 (56.0)	< 0.001
	Tied/clamped cord (not immediately)	34 (75.5)	54 (39.9)		42 (63.8)	< 0.001
	Took mother’s vital signs 15 minutes after birth	17 (23.0)	38 (25.1)		31 (42.8)	0.031
	palpated uterus 15 minutes after delivery	7 (9.1)	26 (21.0)		19 (28.4)	0.041
	Assisted mother to initiate breastfeeding within 1 hour	48 (88.9)	117 (75.6)		58 (81.9)	0.082
**Facility characteristics**						
	Managing authority, government	56 (100.0)	162 (97.8)		8 (12.1)	< 0.001
	Number of delivery couches, M (SD)^b^	10.1 (7.4)	2.2 (1.0)		5.7 (10.5)	0.013
	Deliveries in the past 12 months M (SD)^b^	5,395.4 (1,706.1)	1,459.1 (1,672.5)		1,820.3 (4,672.4)	< 0.001
	Delivery fee, yes	56 (100.0)	155 (98.2)		57 (91.7)	< 0.001
	Piped water, yes	56 (100.0)	138 (86.1)		66 (97.1)	< 0.001
	Central electric supply, yes	56 (100.0)	149 (93.2)		56 (78.9)	0.147
	Maternal and neonatal clinical guideline, yes	34 (74.2)	68 (37.3)		14 (15.2)	< 0.001
**Provider indicators**						
Age M (SD)^b^		34.8 (6.1)	37.9 (7.7)		33.7 (9.3)	0.605
Gender, Male		5 (4.5)	36 (25.1)		11 (15.9)	0.003
Years of experience, M (SD)^b^		7.4 (5.9)	5.5 (6.0)		4.4 (4.3)	0.004
Qualification						
	Specialist/Bsn nurse	7 (7.3)	4 (2.7)		5 (3.8)	< 0.001
	Registered nurse/midwife	43 (86.8)	90 (56.9)		47 (62.8)	
	Enrolled nurse/midwife	6 (5.9)	69 (40.4)		19 (33.4)	
OB/GYN for night duty available, yes		16 (28.7)	14 (9.6)		19 (27.2)	< 0.001
Received incentive						
	No incentive	17 (33.1)	56 (24.8)		29 (29.9)	0.228
	Non-financial only	33 (52.9)	96 (69.0)		32 (58.6)	
	Financial and non-financial	6 (14.0)	11 (6.2)		10 (11.5)	
**Province**						
	Nairobi	-	1 (0.7)		14 (15.8)	< 0.001
	Central	2 (3.1)	28 (18.8)		10 (12.9)	
	Coast	4 (5.0)	22 (10.7)		11 (13.4)	
	Eastern	4 (8.1)	25 (22.5)		5 (13.1)	
	Northeastern	3 (0.0)	13 (2.9)		14 (6.6)	
	Nyanza	12 (20.6)	16 (13.2)		3 (7.5)	
	Rift valley	18 (51.0)	14 (13.4)		7 (21.9)	
	Western	13 (12.2)	44 (17.8)		7 (8.7)	

^a^, Private hospitals include non-governmental organization, private-not-for-profit, private-for-profit, mission and faith-based hospitals

^b^, General linear regression was used to analyze the difference in the continuous indicators while *χ*^2^ was used for the categorical variables; %_wt_, weighted using complex survey method (Taylor series linearization) to adjust for the sampling design; AMTSL, Active management of third stage labor; M (SD), Mean (standard deviation)

#### Determinants of quality of obstetric care

The determinants of quality of care were categorized into three groups: facility, provider, and region. The facility indicators include: the level of health facility, including primary care level (district/sub-district hospitals and health centers), secondary and tertiary care levels (provincial and national hospitals), and private hospitals (non-governmental organizations, private-not-for-profit, private-for-profit, and mission and faith-based hospitals); managing authority (government versus non-government), which could be different than its ownership since some private hospitals were managed and supported by the government, and vice versa; delivery capacity (as indicated by the number of delivery couches); number of deliveries in the past 12 months; whether a delivery fee was administered; availability of piped water; and, availability of central electric supply. The government operated hospitals included most of the health centers, sub-district hospitals, district hospitals, provincial hospitals, and the national hospitals.

Provider characteristics variables include six indicators: age, gender, years of experience, qualification (divided into three categories i.e. specialist/BSN nurse, registered nurse/midwife, and enrolled nurse/midwife), whether obstetrics and gynecologist (OB/GYN) are available for night duty, and whether providers received financial or non-financial incentives (i.e. categorized as no incentives, non-financial incentives only, and both financial/non-financial incentives). Furthermore, region characteristics included eight provinces (i.e. Central, Coast, Eastern, Nairobi, Northeastern, Nyanza, Rift valley, and Western) and 96 districts to account for any remaining influences from locality-specific factors.

### Statistical analyses

We explored characteristics associated with quality of maternity care in three phases–descriptive statistics, negative binomial regression analyses, and finally, structural equation modeling (SEM). For the descriptive statistics, we presented the average performance on quality indicators across the maternity care continuum at different levels of health facility for observations with complete information (*n* = 290). Chi-squared and general linear regression were used to test the differences across different types of facilities for categorical and continuous variables, respectively. A p-value of less than .05 was considered statistically significant.

In the second phase of exploring the determinants of quality at each of the three stages of obstetric care, we combined the dichotomized quality measures into an additive quality indicator, reflecting the count of signal functions offered, respectively for the three stages of care. Negative binomial regression was employed to explore the potential provider and facility determinants. Observations with missing data were also excluded at this phase of our analysis. All regression analyses were weighted using complex survey method (Taylor series linearization) to adjust for the sampling design (see S1 File for programming codes in Stata). Our results were presented as crude and adjusted incidence rate ratios (IRRs). The statistical software for the second phase of the analysis was Stata 13.1 [32].

In the third and final phase of our analysis, we used SEM technique to determine, in addition to determinants of quality, the interrelationship among quality of care in different phases along the care continuum. SEM assessed the latent variables, for example, quality of intrapartum care, using observables measures, such as the seven dichotomized intrapartum care indicators, and it allowed us to examine the determinants of quality and the influences of the quality in an earlier phase on a later phase, all at the same time (see S1 File for SIMPLIS syntax). All of the data (*n* = 627) were used at this stage after imputing missing values.

Under the SEM, we were able to better leverage information in the dataset in the presence of missing data. A modern method for imputation accounting for Missing at Random (MAR) and Missing Completely at Random (MCAR) assumptions, i.e. the Full Information Maximum Likelihood (FIML) method, was employed in LISREL 8.80 to estimate the relationships along the continuum of care for quality maternity care [33]. One benefit of FIML method which employs the Expectation Maximization (EM) imputation technique is the reliable estimation procedure even up to 50% of missing data [29,34,35]. The complex relationships among quality of care for different phases of care, as well as their determinants were presented along with the parameter estimates of the structural model using a path diagram.

## Results

### Descriptive statistics

Table 1 presents the descriptive summary of quality of care indicators and other characteristics by level of health facility. The three categories of health facilities are statistically different at *p* < 0.01 in all the measures of ‘initial assessment and examination’, except for the indicators “washed hand before examination” and “wear sterile gloves for vaginal exam”. Similarly, all measures of ‘newborn and immediate postpartum care’ are statistically at *p* < 0.05, except for the indicator “assisted mother to initiate breastfeeding within 1 hour”. In contrast, the three categories of health facilities are not statistically different at *p* < 0.05 in most of the indicators for ‘intrapartum care’ except “prepared newborn resuscitation equipment” and “assessed integrity of placenta/membranes”.

Meanwhile, the three categories of health facilities differ significantly across geographical distribution and all facility characteristics—from infrastructure (e.g. such water supply), capacity (e.g. number of delivery couches and delivery volume in the past 12 months), to presence of clinical guideline of maternity care, providing evidence to support our categorization of health facilities. Across different types of facilities, providers are not too dissimilar in their age distribution, but their distributions in qualification, experience, and availability for night duty do differ significantly.

### Negative binomial regression analysis

Table 2 presents the crude incidence rate ratios (IRRs) of determinants of quality across the three phases of care. The national/provincial hospitals have higher rates of quality ‘initial assessment and examination’ (IRR = 1.40; 95% CI, 1.21–1.64) and ‘newborn and immediate postpartum care’ (IRR = 1.31; 95% CI, 1.06–1.63) than district/sub-districts hospitals; while the private hospitals had 34%, 14% and 25% higher rates of quality in ‘initial assessment and examination’, ‘intrapartum care’ and ‘newborn and immediate postpartum care’, respectively, than district and sub-districts hospitals. Non-governmental hospitals have a rate 1.10 times higher than government-run hospitals in the quality of ‘intrapartum care’. Hospitals charging delivery fees have higher rates in the quality of ‘initial assessment and examination’ (IRR = 1.37; 95% CI, 1.11–1.68) and ‘intrapartum care’ (IRR = 1.19; 95% CI, 1.10–1.29) than hospitals that are not charging any delivery fee. Meanwhile, facilities with good infrastructure, as measured by piped water are more likely to have better quality during ‘initial assessment and examination’ and ‘intrapartum care’. And the presence of maternal and neonatal clinical guideline is associated with higher quality during initial assessment and examination (IRR = 1.16; 95% CI, 1.01–1.34), and newborn and immediate postpartum care (IRR = 1.26; 95% CI, 1.05–1.52) at *p* < 0.05. Across provider characteristics, the availability of OB/GYN for night duty have a positive impact on quality across all three phases of care. The age and years of experience are also associated with higher rates of quality intrapartum care. Finally, almost all the provinces have lower rates of quality delivery care than Central province across the three phases, except for Nairobi, which has higher rates in the quality of ‘intrapartum care’ (IRR = 1.07; 95% CI, 1.01–1.14) and ‘newborn and immediate postpartum care’ (IRR = 1.25; 95% CI, 1.01–1.53).

**Table 2:** Crude incidence rate ratios (IRRs) of quality of care for intrapartum, newborn and immediate postpartum care.

			Crude incidence rate ratio (IRR, 95% confidence interval)^c^		
Variables			Initial assessment and examination	Intrapartum care	Newborn and immediate postpartum care
**Facility characteristics**					
	Health facility level (Ref: District [sub])				
		National/provincial	1.40 (1.21, 1.64)****	1.06 (0.93, 1.21)	1.31 (1.06, 1.63)**
		Private^a^	1.34 (1.15, 1.55)****	1.14 (1.06, 1.22)****	1.25 (1.02, 1.54)**
	Managing authority (Ref: Government)		1.15 (0.92, 1.43)	1.10 (1.02, 1.18)**	1.06 (0.83, 1.37)
	Number of delivery couches		1.01 (0.99, 1.01)	1.00 (1.00, 1.01)*	1.01 (1.01, 1.02)**
	Deliveries in the past 12 months		1.00 (1.00, 1.00)*	1.00 (1.00, 1.00)	1.00 (0.99, 1.00)
	Delivery fee (Ref: No)		1.37 (1.11, 1.68)***	1.19 (1.10, 1.29)****	1.38 (0.72, 2.66)
	Piped water (Ref: No)		1.21 (1.02, 1.43)**	1.11 (1.04, 1.18)****	1.25 (0.84, 1.86)
	Central electric supply (Ref: No)		1.05 (0.90, 1.22)	0.98 (0.89, 1.08)	0.91 (0.64, 1.27)
	Maternal and neonatal clinical guideline (Ref: No)		1.16 (1.01, 1.34)**	1.02 (0.95, 1.09)	1.26 (1.05, 1.52)**
**Provider indicators**					
	Age		0.99 (0.98, 1.01)	1.01 (1.01, 1.01)**	1.01 (1.01, 1.02)***
	Gender (Ref: Female)		1.16 (0.95, 1.41)	0.99 (0.94, 1.06)	0.97 (0.80, 1.18)
	Years of experience		1.00 (0.99, 1.01)	1.01 (1.01, 1.01)****	1.00 (0.99, 1.01)
	Qualification (Ref: Specialist/Bsn nurse)				
		Registered nurse/midwife	1.01 (0.82, 1.25)	0.94 (0.86, 1.03)	0.83 (0.68, 1.03)*
		Enrolled nurse/midwife	0.99 (0.80, 1.23)	0.94 (0.85, 1.04)	0.86 (0.70, 1.05)
	OB/GYN for night duty (Ref: No)		1.44 (1.26, 1.64)****	1.21 (1.13, 1.29)****	1.42 (1.17, 1.72)****
	Received incentive (Ref: No incentive)				
		Non-financial only	1.02 (0.86, 1.20)	1.01 (0.96, 1.05)	1.03 (0.92, 1.16)
		Financial and non-financial	1.13 (0.96, 1.33)	0.93 (0.85, 1.01)*	0.95 (0.84, 1.07)
**Province (Ref: Central)**					
	Nairobi		0.87 (0.67, 1.12)	1.07 (1.01, 1.14)**	1.25 (1.01, 1.53)**
	Coast		0.68 (0.54, 0.87)***	0.77 (0.71, 0.83)****	0.60 (0.50, 0.73)****
	Eastern		0.65 (0.51, 0.83)****	0.80 (0.73, 0.87)****	0.94 (0.74, 1.19)
	Northeastern		0.62 (0.48, 0.79)****	0.76 (0.70, 0.82)****	0.64 (0.39, 1.02)*
	Nyanza		0.71 (0.50, 1.01)*	0.94 (0.84, 1.04)	0.68 (0.46, 1.00)**
	Rift valley		0.80 (0.69, 0.93)***	0.85 (0.77, 0.94)****	1.05 (0.89, 1.23)
	Western		0.63 (0.52, 0.77)****	0.84 (0.78, 0.91)****	0.59 (0.44, 0.79)***
District^b^			0.99 (0.98, 0.99)**	0.99 (0.98, 0.99)**	0.99 (0.99, 1.00)**

^a^, Private hospitals include non-governmental organization, private-not-for-profit, private-for-profit, mission and faith-based hospitals

^b^, There were a total of 96 districts and was analyzed as a continuous indicator to check for trend

^c^, All analyses were weighted using complex survey method (Taylor series linearization) to adjust for the sampling design

*p < .10

**p < .05

***p < .01

****p < .001

After adjusting for all the variables, national/provincial hospitals still have higher rates of quality care during initial assessment and examination at IRR = 1.54 (95% CI, 1.29–1.85) and IRR = 1.36 (95% CI, 1.04–1.77), respectively, than district/sub-districts hospitals (Table 3). The private hospitals have a higher rate of quality of care in all the three phases of care than district/sub-district hospitals. The increased rates in the quality of ‘initial assessment and examination’ (IRR = 2.57; 95% CI, 1.77–3.71) and ‘intrapartum care’ (IRR = 1.23; 95% CI, 1.11–1.35) associated with delivery fee versus facilities not charging any fee also persists at *p* < 0.05. Likewise, facilities with maternal and neonatal clinical guideline still remain significantly higher rates of quality during ‘initial assessment and examination’ (IRR = 1.30; 95% CI, 1.12–1.52) and ‘newborn and immediate postpartum care’ (IRR = 1.42; 95% CI, 1.22–1.65) at *p* < 0.05 than hospitals without such guidelines. However, the association between provider characteristics and ‘intrapartum care’ is only seen in the years of experience (IRR = 1.01; 95% CI, 1.00–1.01; p = .05), and between age and ‘newborn and immediate postpartum care’ (IRR = 1.01; 95% CI, 1.00–1.02; p = .05). Regional differences are mainly seen in the quality of ‘initial assessment and examination’ with almost all the provinces being at a lower rate of quality of care than Central province. Only Coast province is still statistically significant lower in quality across all three phases of obstetric care.

**Table 3:** Adjusted incidence rate ratios (IRRs) of quality of care for intrapartum, newborn and immediate postpartum care.

			Adjusted incidence rate ratio (IRR, 95% confidence interval)^c^		
Variables			Initial assessment and examination	Intrapartum care	Newborn and immediate postpartum care
**Facility characteristics**					
	Health facility level (Ref: District [sub])				
		National/provincial	1.54 (1.29, 1.85)****	1.06 (0.98, 1.14)	1.36 (1.04, 1.77)**
		Private^a^	2.74 (1.88, 3.98)****	1.22 (1.05, 1.40)***	1.85 (1.29, 2.66)****
	Managing authority (Ref: Government)		0.52 (0.36, 0.76)****	0.90 (0.78, 1.04)	0.65 (0.47, 0.93)**
	Number of delivery couches		0.98 (0.96, 0.99)**	1.00 (0.99, 1.01)	1.00 (0.98, 1.03)
	Deliveries in the past 12 months		1.00 (1.00, 1.00)	1.00 (1.00, 1.00)	1.00 (1.00, 1.00)
	Delivery fee (Ref: No)		2.57 (1.77, 3.71)****	1.23 (1.11, 1.35)****	1.59 (0.93, 2.72)*
	Piped water (Ref: No)		0.94 (0.79, 1.12)	1.09 (1.00, 1.19)**	0.92 (0.64, 1.31)
	Central electric supply (Ref: No)		0.78 (0.64, 0.96)**	0.92 (0.85, 0.99)**	0.86 (0.67, 1.10)
	Maternal and neonatal clinical guideline (Ref: No)		1.30 (1.12, 1.52)****	1.05 (1.00, 1.11)*	1.42 (1.22, 1.65)****
**Provider indicators**					
	Age		1.00 (0.99, 1.01)	1.00 (1.00, 1.00)	1.01 (1.00, 1.02)**
	Gender (Ref: Female)		1.05 (0.90, 1.24)	0.96 (0.91, 1.01)	0.98 (0.86, 1.13)
	Years of experience		1.00 (0.99, 1.01)	1.01 (1.00, 1.01)**	1.00 (0.99, 1.01)
	Qualification (Ref: Specialist/Bsn nurse)				
		Registered nurse/midwife	1.06 (0.83, 1.35)	1.03 (0.93, 1.14)	0.91 (0.72, 1.16)
		Enrolled nurse/midwife	1.15 (0.89, 1.47)	1.02 (0.91, 1.15)	0.92 (0.73, 1.17)
	OB/GYN for night duty (Ref: No)		1.02 (0.86, 1.21)	1.07 (0.98, 1.11)	1.05 (0.85, 1.29)
	Received incentive (Ref: No incentive)				
		Non-financial only	1.06 (0.94, 1.21)	1.04 (0.98, 1.08)	0.98 (0.88, 1.09)
		Financial and non-financial	1.07 (0.86, 1.32)	0.97 (0.90, 1.06)	0.99 (0.84, 1.15)
**Province (Ref: Central)**					
	Nairobi		0.83 (0.61, 1.14)	0.97 (0.87, 1.05)	1.00 (0.73, 1.37)
	Coast		0.42 (0.31, 0.58)****	0.75 (0.65, 0.87)****	0.54 (0.36, 0.81)***
	Eastern		0.43 (0.27, 0.71)****	0.80 (0.64, 1.00)*	1.08 (0.54, 2.14)
	Northeastern		0.72 (0.38, 1.38)	0.91 (0.66, 1.25)	1.18 (0.46, 3.02)
	Nyanza		0.36 (0.17, 0.76)***	1.05 (0.72, 1.54)	0.90 (0.29, 2.82)
	Rift valley		0.34 (0.12, 0.96)**	0.94 (0.58, 1.52)	1.62 (0.36, 7.20)
	Western		0.23 (0.07, 0.77)**	1.04 (0.56, 1.94)	1.02 (0.15, 7.02)
District^b^			1.01 (1.00, 1.03)*	1.00 (0.99, 1.01)	0.99 (0.97, 1.02)

^a^, Private hospitals include non-governmental organization, private-not-for-profit, private-for-profit, mission and faith-based hospitals

^b^, There were a total of 96 districts and was analyzed as a continuous indicator to check for trend

^c^, All analyses were weighted using complex survey method (Taylor series linearization) to adjust for the sampling design

*p < .10

**p < .05

***p < .01

****p < .001

### Structural equation modeling

**Fig 2:**
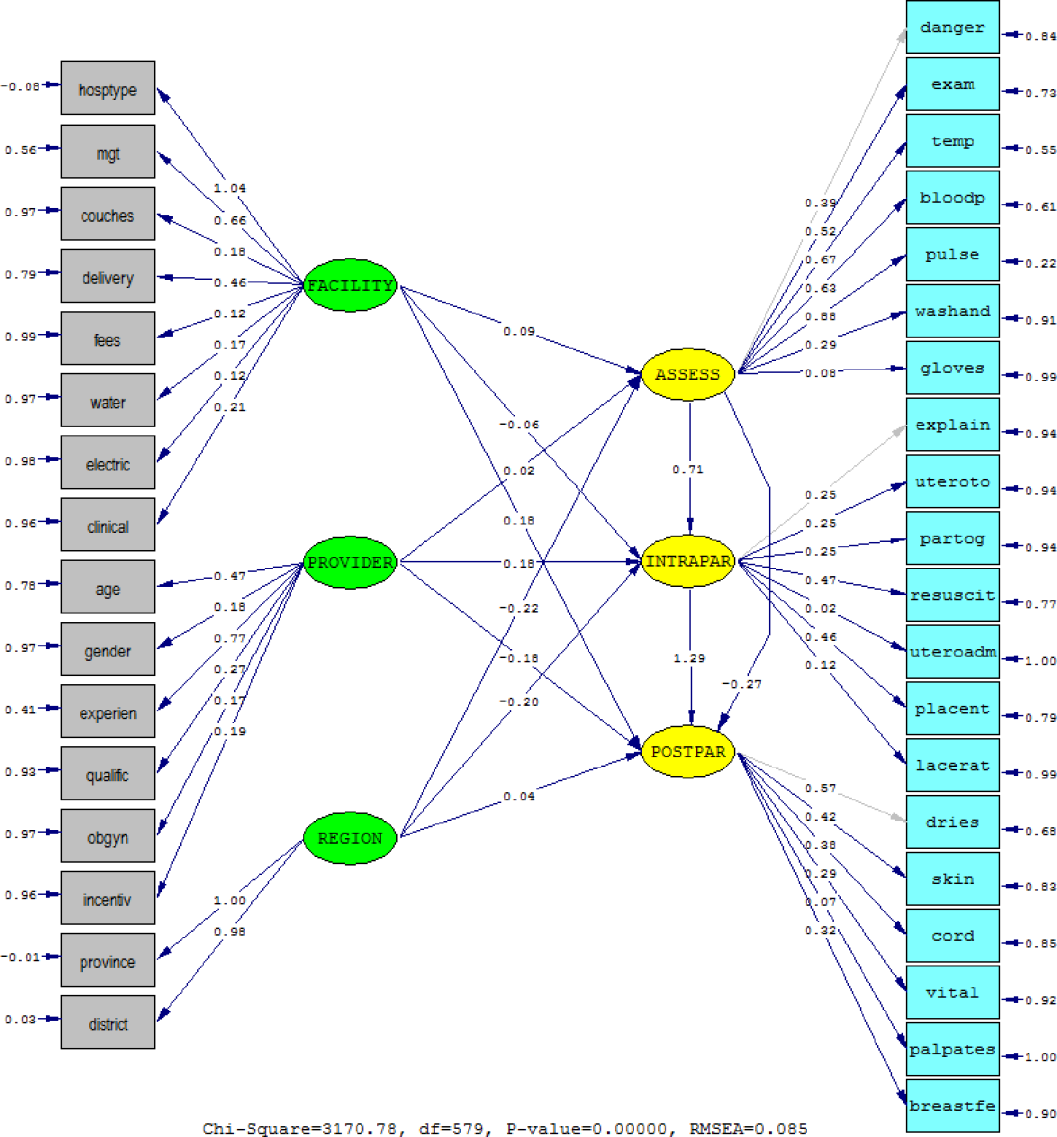
Standardized parameter estimates of the quality of care along the continuum of care for intrapartum, newborn and immediate postpartum care.

The EM algorithm for missing data revealed 112 different missing-value patterns, with the proportion of missing values being 8.04% which were imputed. The path diagram in Fig 2 presents the standardized parameter estimates for quality of care during the assessment, intrapartum, newborn and immediate postpartum care phases. The Root Mean Square Error of Approximation (RMSEA) of 0.085 indicated a marginal goodness-of-fit. Traditionally, RMSEA below 0.08 shows a good fit, and one of its benefit is its insensitivity to sample size unlike the chi-square. All the observed indicators are statistically significant at *p* < 0.05, except for wear sterile gloves for vaginal exam (‘gloves’, *β* = 0.08, *p* = 0.066), correctly administered uterotonic (‘uteroadm’, *β* = 0.02, *p* = 0.667), and palpated uterus 15 minutes after delivery (‘palpates’, *β* = 0.07, *p* = 0.145) (Table 4).

**Table 4:** Standardized parameter estimates of the measurement indicators of the structural model.

		Standardized Parameter estimate (*Se*)	*t*-value
Codes	Variables		
**ASSESS**	**INITIAL ASSESSMENT AND EXAMINATION**		
danger	Asked if experienced any danger sign	0.39 (0.01)	17.15***
exam	Performed general examination	0.52 (0.03)	8.11***
temp	Took temperature	0.67 (0.04)	8.92***
blood	Took blood pressure	0.63 (0.03)	8.72***
pulse	Took pulse	0.88 (0.05)	9.45***
washand	Washed hands before examination	0.29 (0.02)	5.78***
gloves	Wear sterile gloves for vaginal exam	0.08 (0.005)	1.84
**INTRAPAR**	**INTRAPARTUM CARE**		
explain	Explained all procedures	0.25 (0.01)	17.34***
uteroto	Prepared uterotonic drug for AMTSL	0.25 (0.02)	4.01***
partog	Used partograph during labor	0.25 (0.02)	4.05***
resuscit	Prepared newborn resuscitation equipment	0.47 (0.05)	5.09***
uteroadm	Correctly administered uterotonic	0.02 (0.01)	0.43
placent	Assessed integrity of placenta/membranes	0.46 (0.04)	5.05***
lacerat	Assessed for perineal/vaginal lacerations	0.12 (0.004)	2.38*
**POSTPAR**	**NEWBORN AND IMMEDIATE POSTPARTUM CARE**		
dries	Immediately dried baby with towel	0.57 (0.01)	13.72***
skin	Assessed NRE and placed skin-to-skin	0.42 (0.03)	7.82***
cord	Tied/clamped cord (not immediately)	0.38 (0.03)	7.34***
vital	Took mother’s vital signs 15 minutes after birth	0.29 (0.02)	5.73***
palpates	palpated uterus 15 minutes after delivery	0.07 (0.02)	1.46
breastfe	Assisted mother to initiate breastfeeding within 1 hour	0.32 (0.02)	6.29***
**FACILITY**	**FACILITY CHARACTERISTICS**		
hosptype	Health facility level	1.04 (0.02)	28.25***
mgt	Managing authority	0.66 (0.02)	17.19***
couches	Number of delivery couches	0.18 (0.04)	4.75***
delivery	Deliveries in the past 12 months	0.46 (0.06)	11.84***
fees	Delivery fee	0.12 (0.01)	3.16**
water	Piped water	0.17 (0.02)	4.47***
electric	Central electric supply	0.12 (0.01)	3.23**
clinical	Maternal and neonatal clinical guideline	0.21 (0.02)	5.44***
**PROVIDER**	**PROVIDER INDICATORS**		
age	Age	0.47 (0.07)	9.07***
gender	Gender	0.18 (0.02)	3.65***
experien	Years of experience	0.77 (0.08)	11.97***
qualif	Qualification	0.27 (0.03)	5.57***
obgyn	OB/GYN for night duty	0.17 (0.02)	3.47***
incentiv	Received incentives	0.19 (0.03)	3.89***
**REGION**	**REGION**		
province	Province	1.00 (0.07)	34.83***
district	District	0.98 (0.07)	33.45***

*Se*, Standard error

*p ≤ .05

**p ≤ .01

***p ≤ .001 (two-tailed)

**Fig 3:**
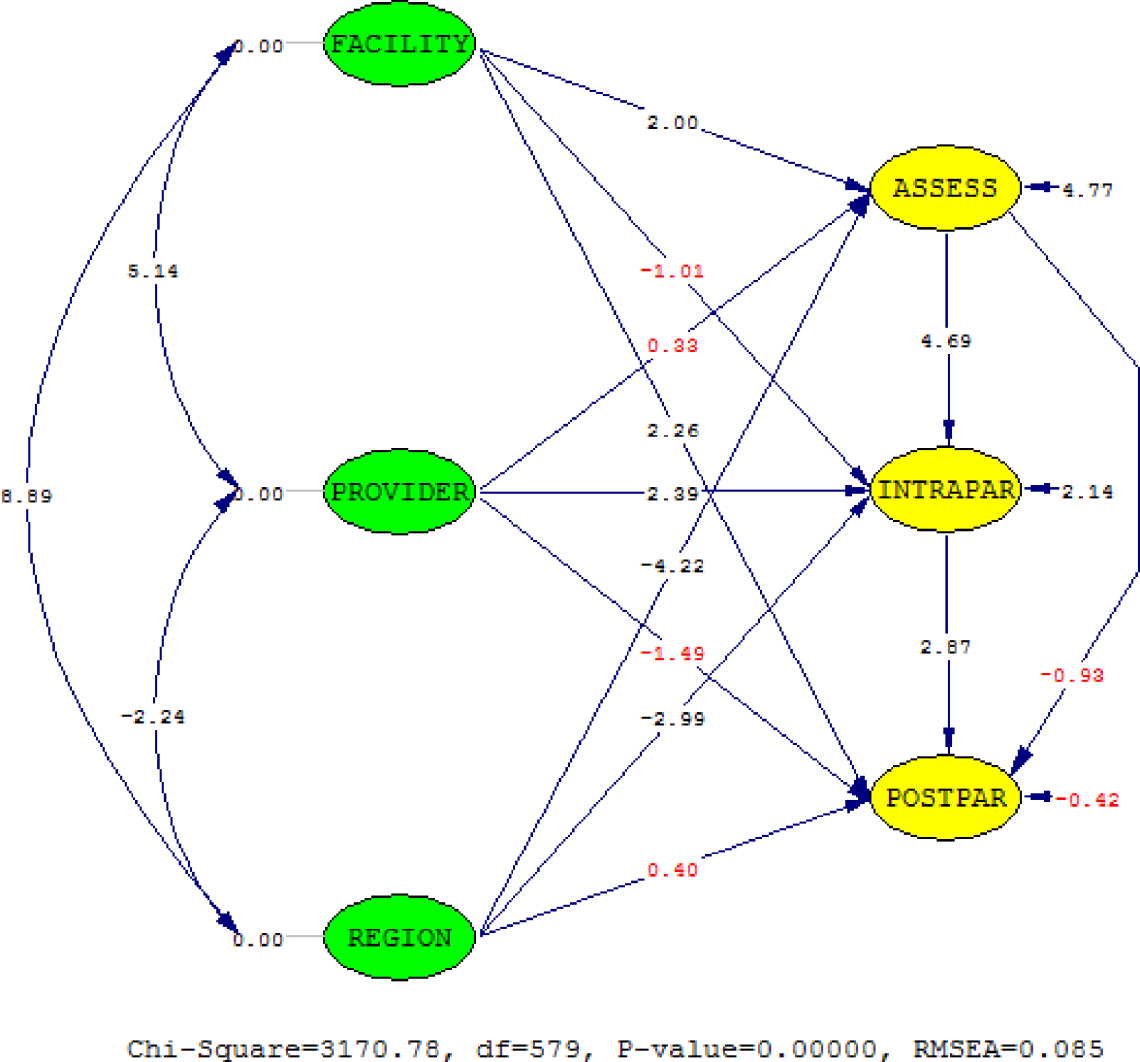
The t-values of the structural path of the continuum of care for intrapartum, newborn and immediate postpartum care. The red values are not significant.

Fig 3 indicates the structural path relationship for quality along the continuum of care, with the numbers shown being t-statistics. The structural path relationship from facility characteristic latent (FACILITY) to initial assessment and examination latent (ASSESS, *β* = 0.09, *p* = 0.046), and to newborn and immediate postpartum care latent (POSTPAR, *β* = 0.18, *p* = 0.024) are positive and statistically significant (Table 5). On the other hand, for provider characteristics latent (PROVIDER), only the path relationship to intrapartum care latent (INTRAPAR, *β* = 0.18, *p* = 0.017) is positive and statistically significant at *p* < 0.05. The regional characteristics latent (REGION) has statistically significant associations with quality during assessment and examination (ASSESS, *β* = -0.22, *p* < 0.001) and intrapartum care (INTRAPAR, *β* = -0.20, *p* = 0.003). Lastly, along the continuum of care, quality of care in the initial assessment have a positive and statistically significant association with quality of intrapartum care (INTRAPAR, *β* = 0.71, *p* < 0.001), which in turn is positively associated with the quality of newborn and immediate postpartum care (POSTPAR, *β* = 1.29, *p* = 0.004).

**Table 5:** Standardized estimates of the path relationships of quality of care along the continuum of care for intrapartum, newborn and immediate postpartum care.

Paths	Parameter^a^	Stdz. Coeff. (Std. Error)	*t*-value
FACILITY → ASSESS	*β*_1_	0.09 (0.05)	2.00*
FACILITY → INTRAPAR	*β*_2_	-0.06 (0.06)	-1.01
FACILITY → POSTPAR	*β*_3_	0.18 (0.08)	2.26*
PROVIDER → ASSESS	*β*_4_	0.02 (0.06)	0.33
PROVIDER → INTRAPAR	*β*_5_	0.18 (0.08)	2.39*
PROVIDER → POSTPAR	*β*_6_	-0.18 (0.12)	-1.49
REGION → ASSESS	*β*_7_	-0.22 (0.05)	-4.22***
REGION → INTRAPAR	*β*_8_	-0.20 (0.07)	-2.99**
REGION → POSTPAR	*β*_9_	0.04 (0.11)	0.40
**ASSESS → INTRAPAR**	***β***_**10**_	**0.71 (0.15)**	4.69***
**ASSESS → POSTPAR**	***β***_**11**_	**-0.27 (0.30)**	**-0.93**
**INTRAPAR → POSTPAR**	***β***_**12**_	**1.29 (0.45)**	2.87**

^a^, The Full Information Maximum Likelihood estimation procedure took into account the missing at random (MAR) and missing completely at random (MCAR) data; Stdz. Coeff., standardized coefficient; Std. Error, standard error

*p < 0·05

**p < 0·01

***p < 0·001 (two-tailed).

## Discussion

The quality of maternity care is critical in improving health and life chances of both mothers and their newborns. Despite of the challenges in measuring quality of care due to its multi-dimensional nature, previous studies have repeatedly demonstrated that there is indeed much to be desired in the obstetric services in LMICs [15,36]. However, little is known about the determinants of their quality, particularly in LMIC, to inform strategies and effective interventions. In this context, our study therefore employs a recently validated index of delivery care quality and explore the determinants across the three phases—initial assessment and examination; intrapartum; and newborn and immediate postpartum. Findings from our regression analyses and the subsequent SEM indicate that quality of care is driven by different sets of factors for different activities over the entire continuum of delivery care.

Traditional analytic approaches on quality of maternity care frequently treat quality of care as either a uni-dimensional notion by using a summary score to indicate the level of quality performance, or a concept with multiple mutually independent dimensions by analyzing the determinants of quality for different categories of activities in separate regression models [17]. The former approach fails to recognize that different health services require different inputs and that measuring quality of maternity care as one score, though operationally appealing, would prevent us from identifying important determinants and hence potential effective interventions specific to certain types of activities. For instance, our SEM model shows that facility characteristics are important determinants of the quality for initial assessment and postpartum care, while characteristics at the provider level become more important in shaping the quality of intrapartum care. If we were to examine the determinants using a single summary score of quality of obstetric care, depending on the relative weights of different phases of care, we could have mistakenly come to the conclusion that only facility or provider determinants matter. Alternatively, we could conclude that neither matters as the effects could be masked through averaging.

Investigating quality of different phases of delivery care with separate analytical models, as we have demonstrated with negative binomial regression analyses in this study, unfortunately only partially addresses the issue. Different phases of delivery care do not have the same set of inputs but nevertheless are provided by the same team of health professionals in the same facility and therefore are highly correlated. Indeed, our SEM models shows that quality of care in one phase of delivery care is positively associated with the quality in the next phase. Failing to account for such correlation of error terms across regressions would lead to consistent but inefficient estimates [37].

The puzzling mismatch between high coverage of essential interventions of maternity care and poor maternal health outcomes underscore the importance of quality in addition to quantity [15]. Yet, as our study shows, there is no single panacea to improving quality of maternity services. Health professionals capable of providing good intrapartum care needs to be complemented with facilities that can enable good services before and after delivery. Meanwhile, our analyses suggest that there are a number of potential interventions that warrant investment to improve quality of care. One such example is the use of maternal and neonatal clinical guideline, with an IRR of 1.30 for initial assessment and examination quality and an IRR of 1.42 for newborn and immediate postpartum care quality. The statistically significant IRR of delivery fees for quality of care before and during delivery, rather than implying to widely implement user fees per se, suggests financial resources available to health facilities are indeed helpful in improving the quality of services in a LMICs setting, particularly for the initial assessment stage where the quality performance more than doubles (IRR = 2.57) with the presence of delivery fees. In recent years, the Kenyan government has been in the process of implementing free maternity care for all women in public hospitals, and hence, whether the quality of care will be further compromised leading to higher turnover rate from public hospitals and increase in out-of-pocket payment as a result of shift to private hospitals is yet to be determined. Meanwhile, we also found that private hospitals performed better than district/sub-district hospitals across all three phases of obstetric care, and the IRRs in were even relatively higher than those of the national/provincial hospitals in the adjusted models. Since majority of the Kenyan population visit public health facilities for health care because of high costs associated with private health facilities, the government should therefore work toward building the capacity of public health sector to ensure quality of care to reduce preventable maternal deaths.

Worth noting, however, is the strong positive relationship from one stage of delivery care to another, that is–the quality of initial assessment is related with the quality of intrapartum care which is subsequently related with quality of newborn and immediate postpartum care. This is important, especially in LMICs where quality of initial assessment may not necessarily mean quality of post-delivery care. A healthy woman may die within hours not because of the poor initial assessment but because of severe bleeding after delivery if she is unattended. Moreover, infection after childbirth may not be eliminated if the quality of care is compromised. A continued focus on quality of care along the continuum of delivery care is therefore important not only to saving the life of mothers but also to giving the newborn a healthy start in life. Policymakers should therefore consider availing necessary resources and also ensure adequate supervision and emphasis on the quality of delivery care.

To our knowledge, this study is one of the first that empirically applies the expert-validated quality index for delivery developed by Tripathi and colleagues [17]. This new quality index allows us to explore the determinants of quality at different phases as well as the relationship among quality across the continuum of care. Nevertheless, our findings are also constrained by the inherent limitations of this index. One example of such is that the index is a process quality measurement focusing on routine care. It is difficult for us to ensure that the quality indicators are also strongly indicative of maternal health outcomes in our sample, nor can we make inferences regarding the determinants of quality for care provided to specific subgroups or rarer events, such as mothers requiring urgent cesarean deliveries. The potential Hawthorne effect is another limitation inherent in our approach to measure quality. Facility and observation-based measurement of quality is particularly advantageous given its objectivity and consistency, and yet there is still the possibility that the presence of observers would influence provider behavior. Second, we were also limited by the data limitations of the SPA dataset. For instance, the objective of our effort was not to understand all delivery circumstances such as deliveries at home because SPA dataset included only facility-based deliveries. We were also not able to adjust for other potentially important factors such as the staffing levels and resource available at health facilities and health conditions of individual clients that would also shape the provision of care, as such information was not collected. Finally, our study, being observational, is limited in our ability to make causality claims. Although we have included as many potential factors that could shape quality of maternity care as our data allow, there are still potential unobservable factors, such as management capability of health facility managers that are left unaccounted for.

Despite the above limitations, our study points out the importance of studying quality of care of maternity services, and perhaps in general, as a continuum of different sets of activities requiring different inputs, instead of collapsing the multi-dimensional quality performance measures into one summary score. This is particularly important in studying the determinants of quality in LMICs where health systems are frequently resource-constrained on multiple fronts, which needs to be distinctively examined. Quality of care in low and middle income countries is an important and yet have unfortunately been an under-studied issue. Future studies similar to our efforts hereof that investigate the distribution of quality in maternity care are urgently needed to accelerate health gains for millions of mothers and children around the world.

## Supporting information

S1 File The Stata codes for negative binomial regression and SIMPLIS syntax for structural equation modeling. (PDF)
