# Supplementary material for: Quality of maternity care and its determinants along the continuum in Kenya: A structural equation modeling analysis
Source: PLoS One. 2017 May 16;12(5):e0177756. doi: 10.1371/journal.pone.0177756 (PMC5433759; doi:10.1371/journal.pone.0177756)
Supplement: S1 File — (PDF) [file pone.0177756.s001.pdf]

1. **STATA CODES**

```
**********************************
*Outcome variables QoC for intrapartum and postpartum care
**********************************

***Initial client assessment and examination
mark goodinitial if !mi(danger, exam, temp, bloodp, pulse, washand, gloves)
egen initial=rowtotal(danger exam temp bloodp pulse washand gloves) if goodinitial
ta initial

***First, second and third stage labor (intrapartum)
mark goodintra if !mi(explain, uteroto, partog, resuscit, uteroadm, placent, lacerat)
egen intrap=rowtotal(explain uteroto partog resuscit uteroadm placent lacerat) if goodintra
ta intrap

***Newborn and immediate postpartum care
mark goodpost if !mi(dries, skin, cord, vital, palpates, breastfe)
egen postp=rowtotal(dries skin cord vital palpates breastfe) if goodpost
ta postp

*********************************
*******TABLE 1*******************
*********************************

******QoC indicators**********
*Provider Weight
gen wt3= c005/1000000 /*client weight*/
svyset [pweight=wt3]

*Initial assessment
foreach var of varlist danger exam temp bloodp pulse washand gloves {
	ta `var' hosptype if good
	svy: ta `var' hosptype if good, percent col
}

*Intrapartum care
foreach var of varlist explain uteroto partog resuscit uteroadm placent lacerat {
	ta `var' hosptype if good
	svy: ta `var' hosptype if good, percent col
}

*Newborn and immediate postpartum care
foreach var of varlist dries skin cord vital palpates breastfe {
```

```
		ta `var' hosptype if good
		svy: ta `var' hosptype if good, percent col
}


********Facility characteristics********

*Facility Weight
gen wt1=v005/1000000
svyset [pweight=wt1]

*facility type
recode c007 (1/2=0 "National/provincial") (3/4 6=1 "District/Sub-district") (5 7=2 "Other Hosp-Private"), gen(hosptype)
ta hosptype if good

*Managing authority
recode c008 (1=0 "govt/local municipality") (2/4=1 "NGO/PNFP/PFP/Mission/faith-based"), gen(mgt)
ta mgt hosptype if good
svy: ta mgt hosptype if good, percent col

*Number of delivery couches
svy: mean v501a if hosptype==0 & good
svy: mean v501a if hosptype==1 & good
svy: mean v501a if hosptype==2 & good
svy: reg v501a hosptype if good
*SD = Se*√n
display .9924867*sqrt(56)
display .0749597*sqrt(163)
display 1.249968*sqrt(71)


*Number of delivery clients past 12 months
svy: mean v516 if hosptype==0 & good
svy: mean v516 if hosptype==1 & good
svy: mean v516 if hosptype==2 & good
svy: reg v516 hosptype if good
*SD = Se*√n
display 227.9859*sqrt(56)
display 131.0006*sqrt(163)
display 554.5061*sqrt(71)

*Fee for delivery
```

```
ta v504 hosptype if good
svy: ta v504 hosptype if good, percent col

*Piped water
recode v531a (0=0) (1/2=1) (8=.)
ta v531a hosptype if good
svy: ta v531a hosptype if good, percent col

*Electricity
recode v121 (0=0 "No electricity") (1=1 "Central supply")(3=1) (2=0 "Other source") (4/6=0),
gen(electricity)
ta electricity hosptype if good
svy: ta electricity hosptype if good, percent col

*Guideline & protocols
recode v537b (0=0) (1/2=1 ) /*maternal & neonatal clinical*/
ta v537b hosptype if good
svy: ta v537b hosptype if good, percent col

********Provider characteristics********

*Provider Weight
gen wt2=u005/1000000
svyset [pweight=wt2]

*Age
mean w102 if hosptype==0 & good
mean w102 if hosptype==1 & good
mean w102 if hosptype==2 & good
svy: reg w102 hosptype if good
*SD = Se*√n
display .819774*sqrt(56)
display .6024553*sqrt(163)
display 1.099613*sqrt(71)

*Gender
ta c022 hosptype  if good
svy: ta c022 hosptype if good, percent col


*YO experience
gen experience=2011-w107
ta experience
```

```
mean experience if hosptype==0 & good
mean experience if hosptype==1 & good
mean experience if hosptype==2 & good
svy: reg experience hosptype if good
*SD = Se*√n
display .7833117*sqrt(56)
display .4702769*sqrt(163)
display .5102719*sqrt(71)

*Qualification
recode c024(1/21=0 "Specialist/Bsn nurse") (22/23=1 "Reg. nurse/midwife")(24/26=2 "Enrol.
nurse/midwife")(27/96=3 "Others"), gen (qualification)
ta qualification hosptype  if good
svy: ta qualification hosptype if good, percent col

*Night duty ob/gyn
ta v538a hosptype if good
svy: ta v538a hosptype if good, percent col

*Incentive
recode w115 (0=0 "No promotion") (1=1 "promotion opport.")(8=0 "DK/Uncertain"),
gen(promotion)
ta promotion
recode w117 (0=0 "No nonmonetary incentive") (1=1 "nonmonetary incentive"),
gen(nonmonetary)
ta nonmonetary
egen nonfinan=rowtotal(promotion nonmonetary)
ta nonfinan
recode nonfinan (0=0 "No nonmonetary incentive") (1/3=1 "nonmonetary incentive"),
gen(nonfinancial)
ta nonfinancial

recode w116 (0=0 "No salary supplement") (1=1 "salary supplement"), gen(salarysup)
ta salarysup

gen incentive=0
replace incentive=2 if salarysup==1 & nonfinancial==1
replace incentive=2 if salarysup==1 & nonfinancial==0
replace incentive=1 if salarysup==0 & nonfinancial==1
ta incentive hosptype if good

svy: ta incentive hosptype if good, percent col

*Region
```

```
ta v001 hosptype if good
svy: ta  v001 hosptype if good, percent col


*********************************
*******TABLE 2*******************
*********************************

*Initial assessment and examination************************
*Model 1 (Unadjusted)
xtset inv_id

xtnbreg initial ib1.hosptype if good [iweight=wt1] , irr vce(robust) pa
xtnbreg initial i.mgt if good [iweight=wt1] , irr vce(robust) pa
xtnbreg initial v501a if good [iweight=wt1] , irr vce(robust) pa
xtnbreg initial v516 if good [iweight=wt1] , irr vce(robust) pa
xtnbreg initial i.v504 if good [iweight=wt1] , irr vce(robust) pa
xtnbreg initial i.v531a if good [iweight=wt1] , irr vce(robust) pa
xtnbreg initial i.electricity if good [iweight=wt1] , irr vce(robust) pa
xtnbreg initial i.v537b if good [iweight=wt1] , irr vce(robust) pa
xtnbreg initial w102 if good [iweight=wt1] , irr vce(robust) pa
xtnbreg initial i.c022 if good [iweight=wt1] , irr vce(robust) pa
xtnbreg initial experience if good [iweight=wt1] , irr vce(robust) pa
xtnbreg initial i.qualification if good [iweight=wt1] , irr vce(robust) pa
xtnbreg initial i.v538a if good [iweight=wt1] , irr vce(robust) pa
xtnbreg initial i.incentive if good [iweight=wt1] , irr vce(robust) pa
xtnbreg initial ib2.v001 if good [iweight=wt1] , irr vce(robust) pa
xtnbreg initial district if good [iweight=wt1] , irr vce(robust) pa

*Intrapartum care*****************************
*Model 1 (Unadjusted)
xtset inv_id

xtnbreg intrap ib1.hosptype if good [iweight=wt1] , irr vce(robust) pa
xtnbreg intrap i.mgt if good [iweight=wt1] , irr vce(robust) pa
xtnbreg intrap v501a if good [iweight=wt1] , irr vce(robust) pa
xtnbreg intrap v516 if good [iweight=wt1] , irr vce(robust) pa
xtnbreg intrap i.v504 if good [iweight=wt1] , irr vce(robust) pa
xtnbreg intrap i.v531a if good [iweight=wt1] , irr vce(robust) pa
xtnbreg intrap i.electricity if good [iweight=wt1] , irr vce(robust) pa
xtnbreg intrap i.v537b if good [iweight=wt1] , irr vce(robust) pa
xtnbreg intrap w102 if good [iweight=wt1] , irr vce(robust) pa
xtnbreg intrap i.c022 if good [iweight=wt1] , irr vce(robust) pa
xtnbreg intrap experience if good [iweight=wt1] , irr vce(robust) pa
```

```
xtnbreg intrap i.qualification if good [iweight=wt1] , irr vce(robust) pa
xtnbreg intrap i.v538a if good [iweight=wt1] , irr vce(robust) pa
xtnbreg intrap i.incentive if good [iweight=wt1] , irr vce(robust) pa
xtnbreg intrap ib2.v001 if good [iweight=wt1] , irr vce(robust) pa
xtnbreg intrap district if good [iweight=wt1] , irr vce(robust) pa

*Newborn and immediate postpartum care*******************************
*Model 1 (Unadjusted)
xtset inv_id

xtnbreg postp ib1.hosptype if good [iweight=wt1] , irr vce(robust) pa
xtnbreg postp i.mgt if good [iweight=wt1] , irr vce(robust) pa
xtnbreg postp v501a if good [iweight=wt1] , irr vce(robust) pa
xtnbreg postp v516 if good [iweight=wt1] , irr vce(robust) pa
xtnbreg postp i.v504 if good [iweight=wt1] , irr vce(robust) pa
xtnbreg postp i.v531a if good [iweight=wt1] , irr vce(robust) pa
xtnbreg postp i.electricity if good [iweight=wt1] , irr vce(robust) pa
xtnbreg postp i.v537b if good [iweight=wt1] , irr vce(robust) pa
xtnbreg postp w102 if good [iweight=wt1] , irr vce(robust) pa
xtnbreg postp i.c022 if good [iweight=wt1] , irr vce(robust) pa
xtnbreg postp experience if good [iweight=wt1] , irr vce(robust) pa
xtnbreg postp i.qualification if good [iweight=wt1] , irr vce(robust) pa
xtnbreg postp i.v538a if good [iweight=wt1] , irr vce(robust) pa
xtnbreg postp i.incentive if good [iweight=wt1] , irr vce(robust) pa
xtnbreg postp ib2.v001 if good [iweight=wt1] , irr vce(robust) pa
xtnbreg postp district if good [iweight=wt1] , irr vce(robust) pa


*********************************
*******TABLE 3*******************
*********************************

*Initial assessment and examination*************************
*Model 2(Adjusted for facility+provider+region)
xtnbreg initial ib1.hosptype i.mgt v501a v516 i.v504 i.v531a i.electricity i.v537b w102 i.c022
experience i.qualification i.v538a i.incentive ib2.v001 district if good [iweight=wt1] , irr
vce(robust) pa

*Intrapartum care******************************
*Model 2(Adjusted for facility+provider+region)
xtnbreg intrap ib1.hosptype i.mgt v501a v516 i.v504 i.v531a i.electricity i.v537b w102 i.c022
experience i.qualification i.v538a i.incentive ib2.v001 district if good [iweight=wt1] , irr
vce(robust) pa
```

```
*Newborn and immediate postpartum care*****************************
*Model 2(Adjusted for facility+provider+region)
xtnbreg postp ib1.hosptype i.mgt v501a v516 i.v504 i.v531a i.electricity i.v537b w102 i.c022
experience i.qualification i.v538a i.incentive ib2.v001 district if good [iweight=wt1] , irr
vce(robust) pa
```

2. **STRUCTURAL EQUATION MODELING (SIMPLIS SYNTAX)**

```
Raw Data from file 'C:\Users\IHP\Desktop\Paper Factory\SPA data - LMICs\1. Analysis
2\SEM\imputed_data.psf'
Latent Variables  ASSESS INTRAPAR POSTPAR FACILITY PROVIDER REGION
Relationships
danger exam temp bloodp pulse washand gloves = ASSESS
explain uteroto partog resuscit uteroadm placent lacerat = INTRAPAR
dries skin cord vital palpates breastfe = POSTPAR
hosptype mgt beds couches delivery fees csection waterp water electric clinical normal emergen
pediatri = FACILITY
age gender experien qualific obgyn medicoff incentiv = PROVIDER
province district = REGION
ASSESS = FACILITY PROVIDER REGION
INTRAPAR = FACILITY PROVIDER REGION ASSESS
POSTPAR = FACILITY PROVIDER REGION ASSESS INTRAPAR
Method of Estimation = Maximum Likelihood
Path Diagram
Wide Print
Print Residuals
Admissibility Check = 25000
Iterations = 25000
End of Problem
```

```
 -------------------------------
                EM Algoritm for missing Data:
 -------------------------------
      Number of different missing-value patterns=     112
      Convergence of EM-algorithm in     6 iterations
      -2 Ln(L) =   136555.54074

      Percentage missing values=   8.04
```
